# The importance of structured noise in the generation of self-organizing tissue patterns through contact-mediated cell–cell signalling

**DOI:** 10.1098/rsif.2010.0488

**Published:** 2010-11-17

**Authors:** Michael Cohen, Buzz Baum, Mark Miodownik

**Affiliations:** 1 CoMPLEX, University College London, London, UK; 2 MRC-LMCB, University College London, London, UK; 3 Physics Department, King's College London, London, UK

**Keywords:** patterning, lateral inhibition, noise, filopodia, signalling, cellular automata

## Abstract

Lateral inhibition provides the basis for a self-organizing patterning system in which distinct cell states emerge from an otherwise uniform field of cells. The development of the microchaete bristle pattern on the notum of the fruitfly, *Drosophila melanogaster*, has long served as a popular model of this process. We recently showed that this bristle pattern depends upon a population of dynamic, basal actin-based filopodia, which span multiple cell diameters. These protrusions establish transient signalling contacts between non-neighbouring cells, generating a type of structured noise that helps to yield a well-ordered and spaced pattern of bristles. Here, we develop a general model of protrusion-based patterning to analyse the role of noise in this process. Using a simple asynchronous cellular automata rule-based model we show that this type of structured noise drives the gradual refinement of lateral inhibition-mediated patterning, as the system moves towards a stable configuration in which cells expressing the inhibitory signal are near-optimally packed. By analysing the effects of introducing thresholds required for signal detection in this model of lateral inhibition, our study shows how filopodia-mediated cell–cell communication can generate complex patterns of spots and stripes, which, in the presence of signalling noise, align themselves across a patterning field. Thus, intermittent protrusion-based signalling has the potential to yield robust self-organizing tissue-wide patterns without the need to invoke diffusion-mediated signalling.

## 1. Introduction

Tissue patterning and morphogenesis must be coordinated during development if groups of cells are to establish self-organizing patterns of gene expression as they divide, change shape and swap neighbours [1]. Few of the mechanisms enabling interacting groups of cells to pattern developing tissues are understood. We recently identified a role for dynamic protrusions in lateral inhibition-mediated patterning of the developing *Drosophila* notum [2]. To gain an insight into the general features of this type of contact-mediated patterning, we present here a computational model of protrusion-based signalling in which we have systematically explored the roles of signalling noise and thresholds. Having previously described a specific role for these protrusions in bristle patterning the developing notum, here, we develop a logical framework in which to analyse the role of noise in pattern formation in general. Using this approach, we have also been able to look at the potential of protrusion-mediated lateral inhibition signalling to generate diverse self-organizing patterns, such as those usually attributed to the action of diffusible morphogens [3] or more complex cell–cell signalling models [4].

Lateral inhibition provides the basis for a self-organizing patterning system in which differentiated cell states emerge from an otherwise uniform field of cells in a diverse range of organisms [5–19].

**Figure 1.**
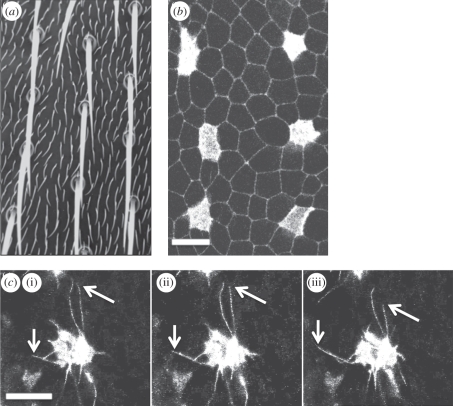
Sensory organ patterning is driven by signalling between dynamic cellular protrusions. (*a*) A section of the notum of an adult *Drosophila melanogaster* fruitfly displays the evenly spaced, grid-like pattern of microchaete bristles that act as mechanosensory organs. Between each bristle are ordinary epithelial cells, each of which expresses a small hair. (*b*) The development of the pattern of mechanosensory organ precursor cells can be observed in fly pupae. The image shows the apical section of epithelial cells in an area of the notum close to the fly midline, at 14 h after pupae formation. Cells destined to become sensory organs (microchaete bristles) express Neuralized-Gal4, UAS-Moesin-GFP (Neu-GFP). Ubiquitously expressed E-Cadherin-GFP is used to visualize apical cell–cell junctions. (*c*) Confocal sections reveal the dynamic protrusions (filopodia and lamelopodia) in the basal section of a typical epithelial cell. The cell in this example is imaged through its expression of Neu-GFP. The image shows the position of filopodia at three 100 s intervals, which extend over multiple cell diameters. The arrows highlight the ends of two filopodia that can be observed extending and retracting (mean filopodia lifetime approx. 500 s—data not shown). Scale bars, 10 µm, (*c*) (i) 0 s, (ii) 100 s, (iii) 200 s.

The basic principle is that identical cells within a two-dimensional epithelial sheet compete to change state. Moreover, cells undergoing a switch in state inhibit their neighbouring cells from doing so. The result of this competition between cells within an emerging patterned field is, over time, the development of a pattern of spaced differentiated cells within the tissue [20,21]. The organization of the mechanosensory organ precursor cells on the notum of the *Drosophila* fly is a good example of a pattern generated in this way [22–25]. During this process, membrane-tethered Delta activates Notch signalling in neighbouring cells, inhibiting their ability to express Delta to yield a spaced pattern of mechanosensory (microchaete) bristles [21,22,24,26] (figure 1*a*,*b*). The need for cell–cell contact in this type of signalling is a key feature of the lateral inhibition process [22,27,28]. Although lateral inhibition signalling systems have been extensively modelled [21,29–32], by quantitatively comparing the results of simulations and experiments, we recently showed that conventional models of Notch–Delta signalling cannot account for bristle spacing on the *Drosophila* notum or the gradual refinement of the pattern of bristle precursor cells, which is observed using live imaging over a period of approximately 8 h in the developing fruitfly notum [2]. In fact, the developing bristle pattern is dependent upon Delta–Notch signalling mediated by dynamic, basal actin-based filopodia that induce intermittent cell–cell signalling contacts between non-neighbouring cells (figure 1*c*). Significantly, these filopodial dynamics generate a type of structured noise that contributes to patterning [2]. We use the terminology *structured noise*, in this context, to distinguish the intermittent signalling arising from a physical process under the control of individual cells, from more stochastic forms of signal noise, such as that which occurs as a result of variations in interactions between proteins in a signal transduction pathway.

The role of noise in creating order in nonlinear dynamical systems is well documented and has been shown to be applicable to understanding chaotic dynamics [33], synchronization [34] and stochastic resonance [35,36]. Living systems are inherently noisy. Moreover, stochastic fluctuations in biochemical processes have been suggested to perform important functions in single cells [37], and within tissues, as subpopulations of identical cells are driven into new cell states as the result of noise inherent in the system [38,39].

To explore the role of signalling noise in patterning, in this paper we have set out to capture the essential elements of protrusion-mediated lateral inhibition patterning using a simple asynchronous cellular automata (CA) model, which lends itself to the analysis of switches between discrete cellular states. A systematic analysis of the effects of signalling noise and thresholds of activation in this model reveals that it is possible to generate a diverse range of patterns incorporating spots and stripes using this type of patterning mechanism. Furthermore, using this general model of pattern formation, we show that, in the presence of signal noise (in the form of oscillations in signal generation or transient breaks in cell–cell communication), stripes align to give well-ordered patterns like those previously attributed to diffusion-based systems [3,40].

## 2. Results

### 2.1. Modelling lateral inhibition using asynchronous cellular automata

**Figure 2.**
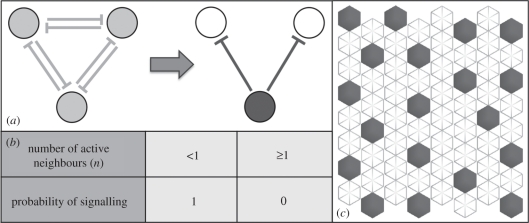
Simulating lateral inhibition patterning. (*a*) A schematic of the lateral inhibition patterning. Initially homogeneous cells (light grey) compete to express an inhibitory signal. Eventually a single cell becomes active (dark grey) and strongly inhibits the expression of the signal in its contacting neighbours. (*b*) The outcome of lateral inhibition signalling expressed as a probabilistic rule set. The signalling probability determines whether a single cell in a field will express an inhibitory signal based on the total number of its active signalling neighbours (*n*). (*c*) An asynchronous cellular automata simulation of lateral inhibition. Cells in the 8 × 8 hexagonally packed array are sequentially selected at random and updated according to the rule table in (*b*). The outcome is a notably disordered packing of active cells (dark grey) expressing the inhibitory signal.

Lateral inhibition patterns arise as a homogeneous group of cells compete to express an inhibitory signal. The end result of this signalling process is cells that either express an inhibitory signal or are inhibited from doing so by signalling cells with which they are in contact (figure 2*a*). In this way, an array of cells can be described as a two-state system, in which cells are either active (expressing inhibitory signals) or inactive (inhibited). This binary state system lends itself to analysis as a two-state CA. Within this formalism, the transition probabilities for lateral inhibition can be easily captured using a simple rule-based logic: a cell with an active neighbour has zero probability of being active, while a cell with no active neighbours has a probability of being active of 1. This is represented in figure 2*b*,*c*, and constitutes a discrete version of the continuum models of lateral inhibition, which rely on threshold concentrations of Notch and Delta determined through coupled differential equations to determine cell state [2,21].

We applied this simple general model of lateral inhibition to a two-dimensional array of hexagonally packed cells to analyse the lateral inhibition process (similar results were generated using other types of packing—see the electronic supplementary material). In this scheme, a cell can be in one of two states: dark grey or white, where a dark-grey cell represents one that actively expresses an inhibitory signal. To simulate the patterning process beginning with a uniform field of white cells, cells in the array were selected at random and updated according to the rule-set described in figure 2*b*. The rules simply state that if a cell has no active signalling neighbours it may actively express an inhibitory signal; if it has one or more active neighbours it may not. A stable pattern quickly emerges using these simple rules to yield a pattern of active (dark grey) cells separated by intervening inactive (white) cells, as shown in figure 2*c*.

### 2.2. Without signal noise, emergent patterns remain fixed and irregular

The emergent lateral inhibition patterns, such as that shown in figure 2*c*, quickly stabilize, at which point no further changes in cell state take place. As a result, the final arrangement of active cells is set according to the order in which they were first randomly selected and may be quite irregular (quantified using the coefficient of variation (CV) of pattern spacing, as defined in figure 4*c*) as long as it fulfils the requirement that no two signalling cells are in direct contact with one another. Although this particular model is highly simplified compared with previous models of lateral inhibition [21,29–31] (which include dynamical descriptions of protein synthesis or gene network interactions), it shares a common feature, in that it generates a pattern of cell states that remains fixed once established. However, as we recently showed by imaging the lateral inhibition in the developing *Drosophila* notum, *in vivo*, the process is accompanied by a gradual process of pattern refinement [2].

### 2.3. Signal noise can be simulated by adapting cell update probabilities

**Figure 3.**
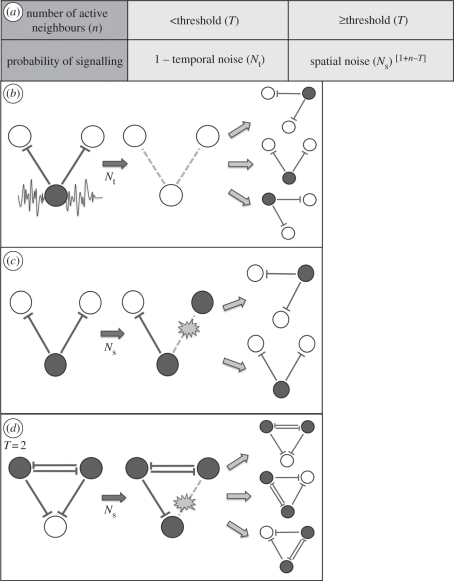
An enhanced model of lateral inhibition incorporating signalling noise and different inhibitory thresholds. (*a*) The rule table determines the probability that a selected cell in an asynchronous cellular automaton will actively express an inhibitory signal. The *threshold* (*T*) is the minimum number of active signalling cells (*n*) required to inhibit an inactive cell. Temporal noise (*N*_t_) is the probability that a cell will stop signalling even without an inhibitory signal at the required threshold. Spatial noise(*N*_s_) is the probability that an inactive cell will signal even when it is in contact with the threshold number of active signalling cells. This probability reduces as the number of active neighbours increases over the threshold. (*b*) A schematic of pattern shifting owing to temporal signalling noise. A cell's inhibitory signal (dark grey) oscillates over time. Its signal effectively ceases such that at subsequent time steps neighbouring cells that were previously inhibited may become active. (*c*) A schematic of pattern shifting owing to spatial signalling noise. A signal ‘connection’ is broken and an inactive cell (white) no longer receives an inhibitory signal and becomes active. At subsequent time steps when the connection is re-established, the cells compete and a stable configuration is re-established. (*d*) A representation of spatial noise in which a minimum threshold of two active cells is required to inhibit a third cell. As in (*c*) the pattern may shift as a result of the signal noise.

Having previously suggested that signalling noise could be involved in this process of refinement, we next used the CA model of lateral inhibition to consider the effect of signalling noise on the long-term development of patterns by introducing non-binary cell update probabilities into the model. These generalized rules are defined in figure 3*a*. Two types of ‘structured’ noise were considered in turn, as follows.

#### 2.3.1. Spatial noise

Spatial noise (*N*_s_) arises as a result of a spatial variance in the strength of cell–cell signals (figure 3*c*), and serves as a simple model of the transient signalling contacts identified in Cohen *et al*. [2] where intermittent cell–cell signalling occurred via dynamic protrusions. This type of noise was implemented in the model by the inclusion of a probability term that defines the likelihood that a selected cell will escape the inhibition of its actively signalling neighbours. It reflects a situation in which the strength of the perceived inhibitory signals are weak enough to allow an inhibited cell to switch state and signal, and therefore scales with the number of signalling neighbours: the more signalling neighbours the lower the chance that sufficient connections are broken to lead to a loss of signal.

In order to fully explore the potential for patterning in this type of signalling system, we generalized the model to account for inhibitory signal thresholds that may occur when a cell has multiple neighbours. Therefore, in the model, a cell is only able to be inhibited from expressing a signal if it is in contact with a set number of actively signalling cells (denoted by a threshold value *T*, figure 3*d*).

#### 2.3.2. Temporal noise

Temporal noise (*N*_t_) arises as a result of fluctuating inhibitory signals generated within each signal-sending cell (figure 3*b*). These fluctuations may emerge in a structured way from oscillations inherent in the signalling protein expression [41–43]. Alternatively it is possible that the signalling pathway is set up in such a way that stochastic variation in the concentration of signalling proteins frequently tips the inhibitory signal above and below the signalling threshold, without the need for controlled oscillations. This type of noise was implemented in the model by the inclusion of a probability term that defines the likelihood that a selected cell will fail to signal to its neighbours even when able to do so. It is equivalent to a situation in which the inhibitory signal put out by a cell drops below some required threshold for long enough that its neighbours may escape the inhibition and change state.

Crucially, both types of signal noise in this model may enable the reconfiguration of a pattern by allowing previously inhibited cells to signal (figure 3).

### 2.4. With signal noise, pattern order increases

**Figure 4.**
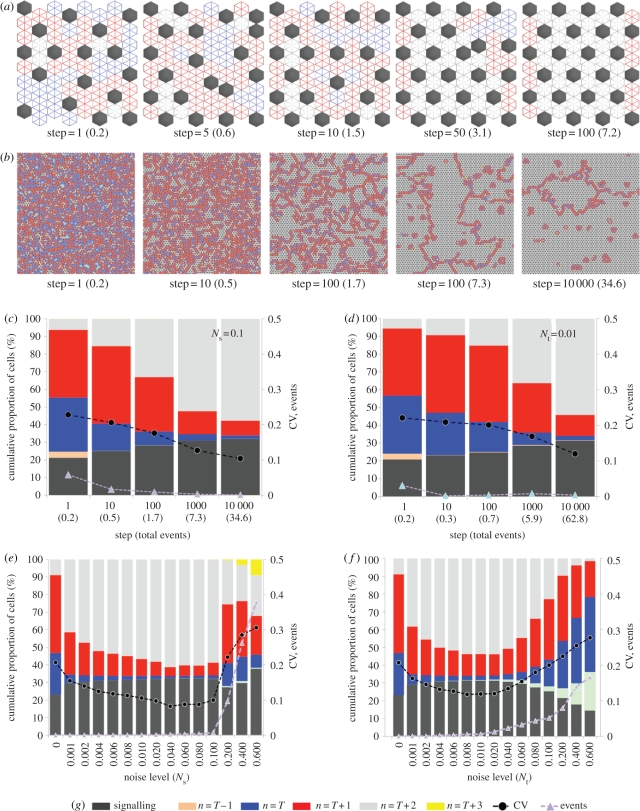
Signal noise leads to pattern optimization. (*a*,*b*) Simulations of inhibitory signalling with spatial noise, *N*_s_ = 0.1, *T* = 1, (*a*) executed in an 8 × 8 hexagonally packed array of cells and (*b*) a 100 × 100 array of cells with toroidal boundaries. Each image shows the pattern at a particular ‘step’ in the simulation advancing from left to right. A single *step* represents a number of cell updates equal to the total number of cells in the array. The number shown in brackets represents the total proportion of cells that have switched state (total events). The state of cells is defined by the colour key in (*g*): dark-grey cells actively express the inhibitory signal and all inactive cells are coloured according to their total number of active neighbours. As the patterns move towards a state of optimized packing this corresponds to a reduction in the number of blue (one active neighbour) and red (two active neighbours) cells and an increase in the number of light-grey cells (three active neighbours). Note that with spatial noise adjacent cells sometimes signal (see steps 5 and 50 in (*a*)), which causes the shift in the pattern towards the optimized state (compare with optimization under temporal noise in the electronic supplementary material, figure S1). In a large field (*b*) this leads to the development of relatively stable optimized ‘zones’ with unstable active boundaries, which expand over time. See also the electronic supplementary material, movie 1. (*c*,*d*) The change over time in the proportion of each cell type represented as a cumulative percentage (plotted on the left-hand *y*-axis). Data are averaged over 10 simulations. In (*c*) the conditions are as described in (*b*). The results of identical simulations with temporal noise, *N*_t_ = 0.01 are shown in (*d*). In addition, the purple triangles show the number of events occurring at each step. The black circles show the coefficient of variation (CV) in the pattern spacing that was measured by recording the distance between each active cell and its six nearest neighbours and taking the ratio of the standard deviation to the mean. The CV and events are plotted on the right-hand *y*-axis. (*e*,*f*) A comparison of the final pattern state achieved after 10 000 steps with different amounts of spatial noise (*e*) and temporal noise (*f*). The figures show the mean values from 10 simulations. Optimized patterns are achieved with noise levels in the ranges 0.001 < *N*_s_ < 0.1 and 0.001 < *N*_t_ < 0.01. At higher levels of noise, the patterns become unstable, as represented by the significant increase in the number of events. NB: Standard errors (95% confidence intervals) in the mean data displayed in (*c*–*f*) were less than 1% (left-hand *y*-axis) and less than 0.01 (right-hand *y*-axis) and so were not visible on this scale.

Next, we tested the impact of signal noise in the CA. Figure 4*a* shows the result of one such simulation with a spatial noise term of *N*_s_ = 0.1 and a threshold *T* = 1. The figure shows the progression of the pattern development from step 1 to 100. (A single step is defined as a number of random selections equal to the number of cells in the array.) An initial pattern of active (dark grey) cells was formed by step 1. At this stage, the pattern is equivalent to that obtained without any noise (as in figure 2*c*). Approximately one in five cells has switched state from inactive to active, as is reflected in the total proportion of cells that have switched state equalling 0.2 (shown in brackets in figure 4). To better study the development of the pattern we labelled inactive cells with different colours representing their number of active neighbours (figure 4*g*). Thus, in the simulation, inactive cells with *T* + 2 active neighbours are coloured light grey, inactive cells with *T* + 1 active neighbours are coloured red and inactive cells with *T* active neighbours are coloured blue.

As the simulation progresses the pattern changes under the influence of spatial noise (as can be observed at time steps 5 and 50). As a result, the pattern of active cells increases in density, and packing order, eventually filling the array in a nearly perfectly optimized arrangement (after 100 steps, an average of 7.2 state changes for every cell in the array). This is accompanied by a corresponding reduction in inactive cells with one (*T*) or two (*T* + 1) active neighbours and an increase in those with three (*T* + 2) active neighbours. These changes reflect the fact that the probability a cell will fail to receive an inhibitory signal becomes steadily reduced as the density of active neighbouring cells increases. We find that, under the influence of signalling noise, there is an inherent tendency for the pattern of signalling cells to become more densely and regularly packed over time.

A similar process of pattern optimization was also observed when we used temporal noise (electronic supplementary material, figure S1). However, in contrast to the spatial noise model, during the patterning process, signalling cells are not observed in adjacent locations. With temporal noise, active cells switch off their signal, thus allowing neighbouring inactive cells to switch state and signal in locations where they were previously inhibited from doing so. With this type of noise, gaps temporarily appear in the pattern. Importantly, although the outcome of both types of noise is almost identical, the dynamics that lead to pattern optimization are distinct.

To test for size effects, we repeated the simulations in a larger field of 100 × 100 cells with toroidal boundary conditions (figures 4*b*; electronic supplementary material, figure S1*b* and movie M1) where the same process of pattern optimization was observed. In this case, zones of perfectly packed active cells emerged surrounded by boundaries of less well-ordered packing (indicated by red and blue cells) where cells continually updated owing to the signal noise. Over time, the perfectly packed zones expanded until the whole field of cells was optimally packed (after approx. 10 000 steps).

To visualize the dynamics of patterning, we plotted the average data recorded from 10 simulations in figure 4*c* (reducing standard errors to less than 1%). This cumulative plot shows the relative proportion of active and inactive cells with different neighbourhoods. It reveals a gradual increase over time in the density of active cells, which is accompanied by a decrease in inactive cells with one or two neighbours and an increase in those with three neighbours. Correspondingly, there is a decrease in the CV of the pattern spacing, which reflects the increased order in the patterning. Finally, figure 4*c* shows that the number of changes in cell states per time step (events) decreases over time. This reflects the fact that the pattern becomes more stable as it becomes more densely packed. Early in the development many cells shift position. Later, the probability of seeing a cell change state is dramatically reduced; generally only occurring in regions that are less well packed.

Figure 4*d* demonstrates that a very similar patterning dynamic can be achieved with temporal noise in place of spatial noise. However, with temporal noise comparably fewer events occur in the early phase of the optimization process and more occur at later steps. This reflects the different event probabilities in each case. Similar dynamics were also observed when both types of noise were implemented at the same time (data not shown).

The same pattern refinement was observed in hexagonally packed arrays with fixed boundaries of odd- and even-sided rectangular dimensions (data not shown), confirming that this phenomenon was not dependent on the particular grid sizes used. We also tested the effect of noise on lateral inhibition carried out in a Voronoi lattice (mimicking a tissue with highly irregular cells; electronic supplementary material, figure S2) and a square lattice of cells (electronic supplementary material, figure S3). In both cases, there was a significant increase in pattern order owing to noise, revealing that this effect is not linked to a specific type of CA lattice. It was also found that, in general, the initial conditions of cells (active or inactive) that were implemented in simulations had little effect on the final pattern. The lateral inhibition patterning rules operate in such a way that a stable configuration will be restored over time.

To test the effect of varying the amount of noise we compared the developed patterns after 10 000 simulation steps with both spatial and temporal noise (figure 4*e*,*f*). For both schemes, a wide range of noise levels gave rise to patterns with increased order compared with the zero noise case. At very low noise levels there was a moderate effect. At intermediate values, the patterns were notably better optimized, as indicated by a higher density of active cells and inactive cells with many active neighbours. The values that gave the most ordered patterns were *N*_s_ = 0.1 and *N*_t_ = 0.01. At higher values of *N*_t_ or *N*_s_, the system became disordered, and failed to form a stable pattern (as reflected in the significantly higher number of events and presence of inactive cells with zero (*T* − 1) neighbours). This result emphasizes that for signal noise to be correctly tuned to aid patterning it must be sufficiently high to enable some cells to reverse their signalling state, particularly in low-density regions of a pattern, but must be sufficiently low that the likelihood of this occurring becomes negligible once a pattern becomes dense and regularly packed. Between these limits noise increases pattern order.

### 2.5. Patterns of spots and stripes can be obtained through signalling with noise at different ranges and thresholds

**Figure 5.**
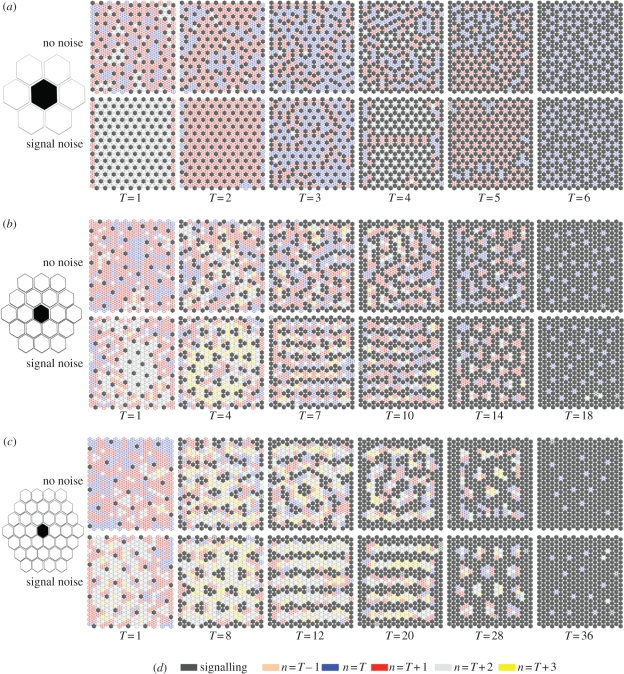
At different signal ranges and inhibitory thresholds, a broad scope of patterns can be achieved. (*a*–*c*) Each panel shows typical results after 1000 steps for simulations of inhibitory signalling carried out in a 20 × 20 hexagonally packed array of cells. Different signal ranges were implemented, as illustrated by the size of the hexagonal shells positioned on the left ((*a*) one cell, (*b*) two cells and (*c*) three cells). For each signal range, a selection of inhibitory thresholds is shown with and without temporal noise, *N*_t_ = 0.01. The active signalling cells and the neighbourhood of inactive cells are identified according to the key in (*d*). It is clear from this illustrative set of examples that different patterns are achievable ranging from spots through to stripes that may be in some cases realigned by the input of signal noise (e.g. (*b*), *T* = 10).

We recently demonstrated that cell–cell signalling can occur at multiple cell ranges as a result of dynamic filopodial protrusions [2]. Thus, we used our model to investigate the effect of signalling at these ranges of intercellular communication with different inhibition thresholds (*T*). We simulated the effect of communication via protrusions by incorporating larger neighbour shells (figure 5) into the model. In particular, we investigated the influence of communication between cells separated by one, two or three cells. Figure 5 shows some typical examples of the types of pattern that were seen with and without the influence of signalling noise. For the nearest-neighbour communication model (figure 5*a*), the emergent patterning is either isolated spots, which optimize their packing under signal noise, or alternatively rings of signalling cells. However, once the signal range extends to two or three cells, a diverse range of spots, clusters, stripes and rings is obtainable (figure 5*b*,*c*). As can be observed, the thickness of the emergent clusters or stripes is dependent on the signal threshold; however, as for the fine-grained patterns of spots, their average separation is fixed according to the signalling range. Notably, when noise is implemented, the stripes tend to align themselves with the cell boundaries.

**Figure 6.**
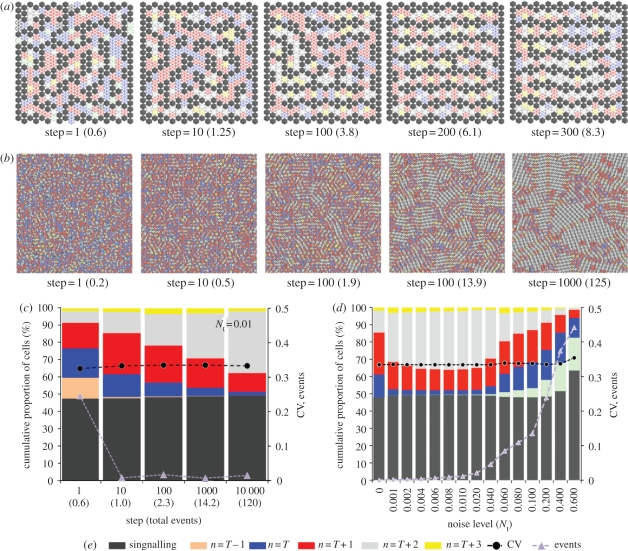
Patterns of stripes align owing to signal noise. (*a*,*b*) Simulations of inhibitory signalling with a signalling range of two cells, an inhibitory threshold, *T* = 9, and temporal signalling noise, *N*_t_ = 0.01. The active signalling cells and the neighbourhood of inactive cells are labelled according to the colour key in (*e*). The number of simulations steps and total events (in brackets) is shown progressing from left to right. In (*a*), where a small array of 20 × 20 cells was used, the initial pattern of randomly orientated stripes can be seen to align, over time, with the array boundaries where there is no signal. In (*b*), where a larger array of 100 × 100 cells (with toroidal boundaries) was used, distinct zones of aligned stripes are formed as a result of the signalling noise. See also the electronic supplementary material, movie 2. (*c*) Graphical visualization of the patterning process. The figure shows the cumulative proportion of each cell type (as defined in the colour key in (*e*)) obtained from data averaged over 10 simulations with the conditions specified in (*b*). The process of stripe alignment correlates with a transition from *n* = *T* and *n* = *T* + 1 cells to *n* = *T* + 2 cells. Also plotted are the number of *events* per time step and the CV in the pattern spacing. With stripe alignment there is no change in the CV of the pattern spacing. (*d*) A comparison of the final pattern state achieved after 10 000 steps with different amounts of signal noise. The figures show the mean values obtained after 10 simulations at a signal range of two cells and a threshold, *T* = 9. Optimized patterns are achieved with noise levels in the range 0.001 < *N*_t_ <0.01. Similar results (data not shown) were obtained when spatial noise was used instead of (or in addition to) temporal noise. NB: Standard errors (95% confidence intervals) in the mean values plotted in (*c*,*d*) were less than 1% (left-hand *y*-axis) and less than 0.01 (right-hand *y*-axis) and so were not visible on this scale.

To further investigate the dynamics of this process, we carried out simulations at different lattice sizes and boundary types. Figure 6 shows the results of when noisy signalling was implemented with second neighbour shell communication and an inhibition threshold of nine signalling cells (see also electronic supplementary material, movie M2). In the smaller array with fixed boundaries (figure 6*a*), the stripes align themselves to the cell boundaries (at which there was no signal). In a large array with toroidal boundaries (figure 6*b*), regions of alignment emerge. This demonstrates that the tendency for stripes to locally align is an inherent property of the system that is not boundary dependent. However, in the smaller array, the fixed boundaries bias the orientation of this local effect. Note that when simulations were implemented in a large array with fixed boundaries (data not shown) stripes close to boundaries aligned with them; however, stripes in the central field aligned themselves in arbitrary directions within distinct regions (as in figure 6*b*). We think it probable that, based upon the scale of most biological patterns [3,40], boundary effects will be significant in most developmental systems (as in figure 6*a*).

The analysis of the patterning dynamics in figure 6*c* reveals that, as with the optimization process observed in figure 4, stripe alignment is concurrent with a significant change in the proportion of inactive cells. During pattern development, there is a steady reduction in the number of cells with *T* or *T* + 1 neighbours and an increase in cells with *T* + 2 neighbours. However, there is no great increase in the active cell density, as was observed for the *T* = 1 system (figure 4*c*). In this case, the active cells reposition themselves in such a way that inactive cells have a greater number of active neighbours. They are, therefore, less likely to experience a lack of inhibitory signalling owing to noise and as a result are less likely to begin signalling and cause the pattern to reposition. Thus, the alignment of stripes represents the most stable pattern configuration in a noisy signalling environment. Figure 6*d* reveals that the range of signal noise for which a stable optimized pattern is achieved is similar to the *T* = 1 case in figure 4*d*. Identical results (not shown) were also achievable with spatial noise. Furthermore, refinement of stripe patterns was also achievable in a square-packed array (electronic supplementary material, figure S3).

## 3. Discussion

The pattern of sensory organs in the notum of *Drosophila* is spaced by a process of inhibitory cell–cell signalling mediated by highly dynamic filopodia [2]. This type of intermittent signalling can be regarded as a type of structured signal noise, for which we have developed a discrete cellular model that is general enough to be applied to many other experimental cellular patterning systems. In this model of lateral inhibition, signal noise was imposed by the inclusion of a probability term that allowed cells to change their state during the patterning process. This was shown to lead to increased order and pattern optimization because, as active signalling cells rearranged themselves into more optimal configurations (dense, ordered fine-grained spots for the *T* = 1 model and aligned stripes for the *T* > 1 models), the probability of a state change occurring was reduced.

The correlation of protrusion length and the separation of spots has been observed in the organization of microchaete bristles on the notum of the adult *Drosophila* fly through the investigation of mutants. Cohen *et al*. [2] have shown that when filopodia length is reduced in the Rac and Scar mutants, the separation of Delta-expressing cells is correspondingly reduced. Here, we have shown a similar effect of changing the cell–cell communication length through changes in the CA nearest neighbour shell.

This study also reveals how striped patterns can be achieved using a discrete model of cell–cell signalling. By allowing signalling to occur at different distances, mediated via filopodial contacts, a diverse range of patterns could be achieved by changing the inhibitory signal threshold. Previous theoretical studies have demonstrated the formation of ‘Turing patterns’ in a discrete cellular system in which the diffusion of two signalling molecules occurs between cells [44,45]. This study identifies a simple mechanism for communication via filopodia that occurs at multiple cell ranges without the need to invoke diffusible morphogens.

The results of our CA lateral inhibition model in the absence of noise are identical to those obtained in previous continuum models of lateral inhibition based on coupled diffusion equations [21,29–31]. Furthermore, with this signalling regime in place, the intermittent signalling presents a simple mechanism by which patterns of stripes are able to align through structured signal noise. This type of stripe alignment is a phenomenon observed in fishes that has previously been modelled as a reaction–diffusion system [46,47] but could be attributable to a cell–cell communication system of the type identified here.

While we have related spatial noise to the filopodial signalling model, we have also shown that temporal noise can lead to pattern optimization in a nearly identical way. Therefore, these results may also have important implications for the understanding of oscillating lateral inhibition-type signals that have been observed in developmental systems [41–43].

Although there have been many exaggerated claims for the beneficial effect of noise in biological systems, particularly in the context of stochastic resonance [35], the effect we have identified here has a very straightforward physical origin that is analogous to the phenomenon of annealing in crystals. Ordered systems, such as crystals, are rarely perfect, and so contain defects (vacancies, dislocations, etc.) [48]. When these defects are allowed to move and find ‘sinks’ (which can be internal crystal boundaries or external crystal surfaces), the perfection of the crystal is increased [49]. The mobility of these defects is dependent on temperature, and so the phenomenon of annealing requires high temperatures (an increase in vibrational noise of the atoms) to reduce defect densities and increase system order. Thus, the solid-state phenomena of grain growth [50] and recrystallization [49], which involve the growth of ‘perfect’ domains of order in a less ordered crystal, can be said to rely on an increase in system noise. We have used the language of defects to graphically illustrate the dynamics of pattern optimization in our system by colouring cells depending on their neighbour states. This gives images of the evolution of our system that show the movement of disorder boundaries (figure 6*b*) that are strikingly similar to grain boundaries in grain growth and recrystallization [49,50]. The concept of simulated annealing [33] in applied mathematics, which involves the simulation of the effects of temperature to find low-energy (ordered) states, is another similar model. These examples show that the phenomenon of noise-induced refinement of patterns is a well-established principle. We propose that our biological model described here is another example of this principle, which because of its generality is likely to have applicability for a wide range of patterning processes in biological tissues.

## 4. Methods

The CA were implemented in C++ . The algorithms used represent a direct implementation of the rule tables defined in figures 2*b* and 3*a*. Cells in the asynchronous automata were picked sequentially at random by implementing a time-seeded random number generator. The state of cells was updated according the rule set, which was based on their number of signalling neighbours and the particular signal threshold being implemented. The number of signalling neighbours was calculated by a summation of active cells in a cell's neighbourhood, whose range was dependent on the particular shell size implemented (1, 2 or 3 cells in the hexagonal array; figure 5). The probabilistic update rules were simulated by generating a random float between 0 and 1: if this was less than the signalling probability (figure 3*a*) then a cell would become active; otherwise it would become inactive.

Simulations executed in the large arrays (100 × 100) were run for a total of 10 000 steps (equivalent to 100 000 000 individual cell selections) with a total run time of approximately 20 min. The graphical output was generated using OpenGL. For the realistic cell topology model (electronic supplementary material, figure S2), cells were generated using the Voronin function in Matlab to generate cell boundaries around a randomly scattered set of points.

## References

[1] Classen A. K. Anderson K. I. Marois E. Eaton S. 2005 Hexagonal packing of *Drosophila* wing epithelial cells by the planar cell polarity pathway. Dev. Cell 9, 805–817 10.1016/j.devcel.2005.10.016 (doi:10.1016/j.devcel.2005.10.016) 16326392

[2] Cohen M. Georgiou M. Stevenson N. L. Miodownik M. Baum B. 2010 Dynamic filopodia drive pattern refinement via intermittent N-Dl signalling. Dev. Cell 19, 78–89 10.1016/j.devcel.2010.06.006 (doi:10.1016/j.devcel.2010.06.006) 20643352

[3] Maini P. K. Baker R. E. Chuong C.-M. 2006 Developmental biology. The Turing model comes of molecular age. Science 314, 1397–1398 10.1126/science.1136396 (doi:10.1126/science.1136396) 17138885 PMC4383235

[4] Webb S. D. Owen M. R. 2004 Oscillations and patterns in spatially discrete models for developmental ligand-receptor interactions. J. Math. Biol. 48, 444–476 10.1007/s00285-003-0247-1 (doi:10.1007/s00285-003-0247-1) 15052506

[5] Amoyel M. Cheng Y. C. Jiang Y.-J. Wilkinson D. G. 2005 Wnt1 regulates neurogenesis and mediates lateral inhibition of boundary cell specification in the zebrafish hindbrain. Development 132, 775–785 10.1242/dev.01616 (doi:10.1242/dev.01616) 15659486

[6] Baker N. E. Yu S. Han D. 1996 Evolution of proneural atonal expression during distinct regulatory phases in the developing *Drosophila* eye. Curr. Biol. 6, 1290–1301 10.1016/S0960-9822(02)70715-X (doi:10.1016/S0960-9822(02)70715-X) 8939576

[7] Bender L. B. Kooh P. J. Muskavitch M. A. T. 1993 Complex function and expression of Delta during *Drosophila* oogenesis. Genetics 133, 967–978 8462854 10.1093/genetics/133.4.967 PMC1205413

[8] Cobellis L. Caprio F. Trabucco E. Mastrogiacomo A. Coppola G. Manente L. Colacurci N. De Falco M. De Luca A. 2008 The pattern of expression of Notch protein members in normal and pathological endometrium. J. Anat. 213, 464–472 10.1111/j.1469-7580.2008.00963.x (doi:10.1111/j.1469-7580.2008.00963.x) 18691378 PMC2644773

[9] Gerhardt H. 2003 VEGF guides angiogenic sprouting utilizing endothelial tip cell filopodia. J. Cell. Biol. 161, 1163–1177 10.1083/jcb.200302047 (doi:10.1083/jcb.200302047) 12810700 PMC2172999

[10] High F. A. Epstein J. A. 2008 The multifaceted role of Notch in cardiac development and disease. Nat. Rev. Genet. 9, 49–61 10.1038/nrg2279 (doi:10.1038/nrg2279) 18071321

[11] Lewis A. K. Frantz G. D. Carpenter D. A. de Sauvage F. J. Gao W. Q. 1998 Distinct expression patterns of notch family receptors and ligands during development of the mammalian inner ear. Mech. Dev. 78, 159–163 10.1016/S0925-4773(98)00165-8 (doi:10.1016/S0925-4773(98)00165-8) 9858718

[12] Lewis J. 1998 Notch signalling and the control of cell fate choices in vertebrates. Semin. Cell. Dev. Biol. 9, 583–589 10.1006/scdb.1998.0266 (doi:10.1006/scdb.1998.0266) 9892564

[13] Ma E. Y. Rubel E. W. Raible D. W. 2008 Notch signaling regulates the extent of hair cell regeneration in the zebrafish lateral line. J. Neurosci. 28, 2261–2273 10.1523/JNEUROSCI.4372-07.2008 (doi:10.1523/JNEUROSCI.4372-07.2008) 18305259 PMC6671837

[14] Munteanu A. Sole R. V. 2008 Neutrality and robustness in evo-devo: emergence of lateral inhibition. PLoS Comput. Biol. 4, e1000226 10.1371/journal.pcbi.1000226 (doi:10.1371/journal.pcbi.1000226) 19023404 PMC2577890

[15] Murtaugh L. C. Stanger B. Z. Kwan K. M. Melton D. A. 2003 Notch signaling controls multiple steps of pancreatic differentiation. Proc. Natl Acad. Sci. USA 100, 14 920–14 925 10.1073/pnas.2436557100 (doi:10.1073/pnas.2436557100) PMC299853 14657333

[16] Noramly S. Morgan B. A. 1998 BMPs mediate lateral inhibition at successive stages in feather tract development. Development 125, 3775–3787 9729486 10.1242/dev.125.19.3775

[17] Reed R. D. 2004 Evidence for Notch-mediated lateral inhibition in organizing butterfly wing scales. Dev. Genes Evol. 214, 43–46 10.1007/s00427-003-0366-0 (doi:10.1007/s00427-003-0366-0) 14618402

[18] Schellmann S. Schnittger A. Kirik V. Wada T. Okada K. Beermann A. Thumfahrt J. Jürgens G. Hülskamp M. 2002 TRIPTYCHON and CAPRICE mediate lateral inhibition during trichome and root hair patterning in Arabidopsis. EMBO J. 21, 5036–5046 10.1093/emboj/cdf524 (doi:10.1093/emboj/cdf524) 12356720 PMC129046

[19] Wang M. Sternberg P. W. 2001 Pattern formation during *C. elegans* vulval induction. Curr. Top. Dev. Biol. 51, 189–220 10.1016/S0070-2153(01)51006-6 (doi:10.1016/S0070-2153(01)51006-6) 11236714

[20] Bray S. J. 2006 Notch signalling: a simple pathway becomes complex. Nat. Rev. Mol. Cell. Biol. 7, 678–689 10.1038/nrm2009 (doi:10.1038/nrm2009) 16921404

[21] Collier J. R. Monk N. A. Maini P. K. Lewis J. H. 1996 Pattern formation by lateral inhibition with feedback: a mathematical model of delta–notch intercellular signalling. J. Theor. Biol. 183, 429–446 10.1006/jtbi.1996.0233 (doi:10.1006/jtbi.1996.0233) 9015458

[22] Artavanis-Tsakonas S. Rand M. D. Lake R. J. 1999 Notch signaling: cell fate control and signal integration in development. Science 284, 770–776 10.1126/science.284.5415.770 (doi:10.1126/science.284.5415.770) 10221902

[23] Simpson P. Woehl R. Usui K. 1999 The development and evolution of bristle patterns in Diptera. Development 126, 1349–1364 10068629 10.1242/dev.126.7.1349

[24] Usui K. Kimura K. 1993 Sequential emergence of the evenly spaced microchaetes on the notum of *Drosophila*. Dev. Biol. 203, 151–158 10.1007/BF00365054 28305732

[25] Wigglesworth V. B. 1940 Local and general factors in the development of ‘pattern’ in *Rhodnius prolixus*. J. Exp. Biol. 17, 180–200

[26] Heitzler P. Simpson P. 1991 The choice of cell fate in the epidermis of Drosophila. Cell 64, 1083–1092 10.1016/0092-8674(91)90263-X (doi:10.1016/0092-8674(91)90263-X) 2004417

[27] Ahimou F. Mok L. Bardot B. 2004 The adhesion force of Notch with Delta and the rate of Notch signaling. J. Cell Biol. 167, 1217–1229 10.1083/jcb.200407100 (doi:10.1083/jcb.200407100) 15611340 PMC2172611

[28] Sun X. Artavanis-Tsakonas S. 1997 Secreted forms of DELTA and SERRATE define antagonists of Notch signaling in Drosophila. Development 124, 3439–3448 9310338 10.1242/dev.124.17.3439

[29] Ghosh R. Tomlin C. 2001 Lateral inhibition through Delta–Notch signaling: a piecewise affine hybrid model. LNCS 2034, 232–246

[30] Hwang I. Balakrishnan H. Ghosh R. Tomlin C. J. 2002 Reachability analysis of Delta–Notch lateral inhibition using predicate abstraction. In Proc. Int. Conf. High Performance Computing, Bangalore, India, 18–21 December 2002, Berlin, Germany: Springer-Verlag

[31] Marnellos G. Deblandre G. Mjolsness E. Kintner C. 2000 Delta–Notch lateral inhibitory patterning in the emergence of ciliated cells in Xenopus: experimental observations and a gene network model. Pac. Symp. Biocomput. 5, 329–340 10902181 10.1142/9789814447331_0031

[32] Podgorski G. J. Bansal M. Flann N. S. 2007 Regular mosaic pattern development: a study of the interplay between lateral inhibition, apoptosis and differential adhesion. Theor. Biol. Med. Model 4, 43 10.1186/1742-4682-4-43 (doi:10.1186/1742-4682-4-43) 17974031 PMC2203995

[33] Matsumoto K. Tsuda I. 1983 Noise-induced order. J. Stat. Phys. 31, 87–106 10.1007/BF01010923 (doi:10.1007/BF01010923)

[34] Pikovsky A. S. 1992 Discrete model of spatially mixing system. Phys. Lett. A 168, 276–279 10.1016/0375-9601(92)91131-A (doi:10.1016/0375-9601(92)91131-A)

[35] McDonnell M. D. Abbott D. 2009 What is stochastic resonance? Definitions, misconceptions, debates, and its relevance to biology. Plos Comput. Biol. 5, e1000348 10.1371/journal.pcbi.1000348 (doi:10.1371/journal.pcbi.1000348) 19562010 PMC2660436

[36] Moss F. Pierson D. Gorman D. Ó. 1994 Stochastic resonance: tutorial and update. Int. J. Bifurcation Chaos 4, 1383–1397 10.1142/S0218127494001118 (doi:10.1142/S0218127494001118)

[37] Hecht I. Kessler D. A. Levine H. 2010 Transient localized patterns in noise-driven reaction–diffusion systems. Phys. Rev. Lett. 104, 158301 10.1103/PhysRevLett.104.158301 (doi:10.1103/PhysRevLett.104.158301) 20482022 PMC2882887

[38] Singh A. Weinberger L. S. 2009 Stochastic gene expression as a molecular switch for viral latency. Curr. Opin. Microbiol. 12, 460–466 10.1016/j.mib.2009.06.016 (doi:10.1016/j.mib.2009.06.016) 19595626 PMC2760832

[39] Suel G. M. Kulkarni R. P. Dworkin J. Garcia-Ojalvo J. Elowitz M. B. 2007 Tunability and noise dependence in differentiation dynamics. Science 315, 1716–1719 10.1126/science.1137455 (doi:10.1126/science.1137455) 17379809

[40] Murray J. D. 1993 Mathematical biology. Berlin, Germany: Springer

[41] Aulehla A. Pourquie O. 2008 Oscillating signaling pathways during embryonic development. Curr. Opin. Cell. Biol. 20, 632–637 10.1016/j.ceb.2008.09.002 (doi:10.1016/j.ceb.2008.09.002) 18845254

[42] Kageyama R. Niwa Y. Shimoja H. 2009 Rhythmic gene expression in somite formation and neural development. Mol. Cells 27, 497–502 10.1007/s10059-009-0068-1 (doi:10.1007/s10059-009-0068-1) 19466597

[43] Shimojo H. Ohtsuka T. Kageyama R. 2008 Oscillations in notch signaling regulate maintenance of neural progenitors. Neuron 58, 52–64 10.1016/j.neuron.2008.02.014 (doi:10.1016/j.neuron.2008.02.014) 18400163

[44] Levine H. Rappel W. J. 2005 Membrane-bound Turing patterns. Phys. Rev. E Stat. Nonlin. Soft Matter Phys. 72, 061 912 10.1103/PhysRevE.72.061912 16485979

[45] Plahte E. 2001 Pattern formation in discrete cell lattices. J. Math. Biol. 43, 411–445 10.1007/s002850100105 (doi:10.1007/s002850100105) 11767205

[46] Kondo S. Asai R. 1995 A reaction–diffusion wave on the skin of the marine angelfish pomacanthus. Nature 376, 765–768 10.1038/376765a0 (doi:10.1038/376765a0) 24547605

[47] Kondo S. Iwashita M. Yamaguchi M. 2009 How animals get their skin patterns: fish pigment pattern as a live Turing wave. Int. J. Dev. Biol. 53, 851–856 10.1387/ijdb.072502sk (doi:10.1387/ijdb.072502sk) 19557690

[48] Miodownik M. A. 2001 Grain growth, uniform. In Encyclopedia of materials: science and technology, vol. 4, pp. 3541–3636 Amsterdam, The Netherlands: Elsevier

[49] Miodownik M. A. 2009 Monte Carlo models for grain growth and recrystallisation. In ASM handbook series, modelling and simulation: processing of metalilic materials, vol. 22A, article 3J. Materials Park, OH: ASM International

[50] Miodownik M. A. Smereka P. Srolovitz D. J. Holm E. A. 2001 Scaling of dislocation cell structures: diffusion in orientation space. Proc. R. Soc. Lond. A 457, 1807–1819 10.1098/rspa.2001.0794 (doi:10.1098/rspa.2001.0794)

